# No-reference image quality assessment based on global awareness

**DOI:** 10.1371/journal.pone.0310206

**Published:** 2024-10-07

**Authors:** Zhigang Hu, Gege Yang, Zhe Du, Xiaodong Huang, Pujing Zhang, Dechun Liu

**Affiliations:** 1 School of Medical Technology and Engineering, Henan University of Science and Technology, Luoyang, China; 2 Henan Engineering Research Center of Digital Pathology and Artificial Intelligence Diagnosis, Luoyang, China; 3 The First Affiliated Hospital of Henan University of Science and Technology, Luoyang, China; University of the Punjab, PAKISTAN

## Abstract

In the field of computer vision, the application of hand-crafted as well as computer-learning-based methods in the field of image quality assessment has yielded remarkable results. However, in the field of no-reference image distortion, it is still challenging to accurately perceive and determine the quality of an image. To address the difficulties of Image Quality Assessment (IQA) in the field of authentic distorted images, we consider the use of the Swin Transformer (ST) to extract features. To enable the model to focus on both spatial and channel information of features, we design a plug-and-play Global Self-Attention Block (GSAB). At the same time, we introduce a Transformer block in the model to enhance the model’s ability to capture long-range dependencies. Finally, we derive the prediction of image quality scores through a Dual-Branching structure. Our method is experimented on four synthetic datasets as well as two authentic datasets, and the results of the experiments are weighted according to the size of the datasets, and the results show that our method outperforms all the current methods and works well in the Generalization ability test, which proves that our method has a good generalization ability. The code will be posted subsequently at https://github.com/yanggege-new/NR-IQA-based-on-global-awareness.

## Introduction

IQA is a measure of the visual quality of an image. According to the presence or absence of reference images, IQA can be categorized into Full-Reference (FR), Reduced-Reference (RR), and No-Reference (NR) [1]. FR: In the FR problem, not only the distorted image is given but also the distortion-free reference image; RR: Distorted images are given, the original distortion-free image is not given, but some information about the distortion-free reference image is given; NR: Only the distorted image is given, without any of the rest of the information given. Therefore, NR is the most difficult image quality assessment. Common types of image distortion are image compression, image blurring, image noise, image brightness and contrast distortion, image cropping and rotation distortion, image overexposure and underexposure, etc. When bad-quality images are used or uploaded to public platforms, they can lead to problems such as distortion of information delivery, degradation of user’s visual experience, visual fatigue, etc., and low-quality images can sometimes hinder the normal self-driving car [2, 3] operation. Therefore, accurate prediction and perception of image quality are crucial for daily life as well as industrial development [4].

According to whether there is human participation, image assessment can be divided into image subjective quality assessment and image objective quality assessment. The subjective quality assessment relies on human subjective feeling to judge the quality of the image, and there are two kinds of assessment indexes: Mean Opinion Score (MOS) (a weighted average of the quality scores given by all the evaluators, the smaller the value, the more serious the distortion, the worse the quality, and the bigger the score, the better the quality), Differential Mean Opinion Score (DMOS) (consider the average score difference between distortion and original content, the larger the score the more serious the distortion, the worse the quality, the range varies with different databases, commonly [0,1], [0,5], [0,9], [0,80], [0,100]). Objective image quality evaluation requires the use of computers to design computational models that can accurately and automatically perceive image quality, test the performance of multiple factors affecting image quality, obtain quantitative values of image quality through the computational models, and then determine the accuracy of the objective evaluation method by comparing it to the subjective human eye observations of MOS or DMOS values. so that the objective assessment methods need to fully incorporate the characteristics of the Human Vision System (HVS). In recent years, with the development of biological sciences, anatomy, neurophysiology, and other disciplines, human understanding of the visual system has gradually deepened. The subjective assessment of the quality of distorted images using manual methods is characterized by low efficiency and high cost, and it is difficult to realize accurate and real-time quality assessment, so it is an inevitable trend to study objective image quality assessment algorithms.

The goal of the no-reference image quality assessment (NR-IQA) or blind image quality assessment (BIQA) methods is to provide a solution when the reference image is not available [5–8]. It has a wide range of applications because no original reference image is required. Deep learning-based image quality evaluation methods typically utilize deep learning structures such as convolutional neural networks (CNNs) to model the perceived quality of an image by learning a large amount of image data so that their predicted image quality scores are highly correlated with the subjective evaluation (MOS or DMOS). There are mainly six existing IQA public datasets, which can be categorized into two kinds, namely, synthetic datasets and authentic datasets, the synthetic datasets are those generated from reference images through various operations such as adding noise or compression in the laboratory, and the authentic datasets are those obtained from natural images taken in real outdoor locations. The synthetic image databases are the LIVE [9] dataset, TID2013 [10] dataset established in 2013, CSIQ [11] dataset, and KADID [12] dataset; and the authentic IQA databases are LIVE Challenge [13] and KonIQ-10k [14] dataset. In this paper, we conduct experiments on the above six datasets to validate the effectiveness of our proposed approach, and finally, the weighting operation is carried out according to the size of the dataset to get the final results, which show that our algorithm outperforms all the current algorithms.

Existing machine learning as well as deep learning methods pay more attention to the local features of an image, but the human eye usually observes the global features before observing the local detail information when observing an image, which makes the machine learning methods miss more important information of the image. Considering the inadequacy of existing methods that focus more on local features during the training process, we propose a no-reference image quality assessment network based on ST, which consists of four parts: the feature extraction block of ST [15], the Transformer Block (TB) for Enhanced Feature Extraction Capabilities, the GSAB [16], and a two-branch structure [17] for quality prediction based on the TB. We first extract the features of the image from ST, which enables the model to process large-size images more efficiently by using a local windowed self-attention mechanism, which helps to deal with the hierarchical structure of the image and multi-scale information. The features extracted by ST are then passed into the GSAB and the TB to enable the model to establish the dependencies between each feature layer. In order to fully utilize the information from different feature layers, we perform multi-scale feature fusion. Unlike the common fusion approach, we use spatial dimension instead of channel feature fusion, and finally the fused features are input into the two-branch quality prediction structure based on TB to predict image quality scores. The contributions of this work are as follows:

- To improve the evaluation of NR images, we design novel spatial feature fusion methods and propose an end-to-end deep learning model.
- We propose a plug-and-play global self-attention module to enable the model to capture rich information between spatial and channel dimensions in the feature map simultaneously.
- We design a TB based dual-branching structure for quality prediction, consisting of two branches: score prediction and weight prediction, and we consider that the final image prediction score depends on the balance between each patch score and weight branch.
- We weigh the final experimental results according to the dataset size, and the experimental results show that our method not only performs well on synthetic datasets, but also achieves good results on authentic datasets, and the SRCC and PLCC of our method exceed the existing methods after weighted averaging. It achieves significant results in the Generalization ability test, which also proves the strong generalization ability of our model.

## Related work

### No reference image quality assessment

NR-IQA refers to the prediction of the quality of a distorted image without reference to the original image or in the absence of the original image. Since no reference image is required, NR-IQA is more widely used in real-life applications. In [18] a method for reference-free image quality assessment specifically for images captured by smartphones is applied, which can help users to improve the quality of the captured images and improve the user experience. In [19], the application of reference-free image quality assessment in medical images is investigated, aiming to ensure that the clarity and quality of images meet the diagnostic standards in medical imaging diagnosis, so as to improve the diagnostic accuracy. In [20] it was studied that a reference-free image quality assessment method was proposed for assessing the image quality of tropical timber. The method accurately evaluates the quality of timber images without reference images and provides an effective tool for timber classification and quality control. The methods applied in NR-IQA are mainly based on Natural Scene Statistics (NSS) [5, 6, 8, 21–26] and learning-based methods [27–32]. NSS focuses on the fact that high-quality natural images will obey certain statistical properties, while low-quality natural images will deviate from these properties. A typical approach to determining image quality using NSS is divided into three key steps: feature extraction, NSS modeling, and feature regression. Features can be extracted from the spatial or transform domain but most of them are manually extracted features. Parametric models such as Generalized Gaussian Distribution (GGD), Multivariate Generalized Gaussian Distribution (MGGD), and Asymmetric Generalized Gaussian Distribution (AGGD) are used in the NSS modeling step. Finally, the parameters of the NSS model are regressed to get the final quality using Support Vector Machine (SVM) and Support Vector Regression (SVR). In [33] for handcrafted features, the results are better on laboratory synthesized images but less capable on authentic distorted images. Learning-based methods mainly include both machine learning and deep learning. However, in previous IQA studies, the convolutional neural network method in deep learning was mainly used, Convolutional neural network (CNN) based methods can give better results in predicting authentic distorted images by extracting features directly from low-quality images [34–43].

### Deep learning for NR-IQA

Deep learning has been widely applied in the field of IQA: quality assessment of optical remote sensing images [44] utilizes CNN and SVM to recognize objects, demonstrating its high accuracy and robustness; shallow CNN avoids the complexity and overfitting problems of deep networks in the clarity assessment of reference-free images, and [45] combines image processing and machine learning techniques to achieve efficient fuzzy detection; [46] improves the quality assessment of optical remote sensing images by integrally considering the image details, structure and color features, the quality assessment of multi-exposure fused images was improved; [47] proposed a standardized process to improve the reproducibility of visual quality assessment of deep features; [48] proposed an assessment method to analyze the inter- and intra-region similarity of images, which outperforms the traditional methods; [20] assessed the quality of tropical timber images by using deep learning techniques; and [49] guided the assessment of image quality through a deep learning network that improves the single-image super-resolution effect. This also proves that IQA has important potential and value in practical applications such as remote sensing, pathology, wood classification and image super-resolution. There are several main methods for predicting image quality that have been used most recently: Hyper-IQA [39] is an NR-IQA technique implemented through an adaptive hypernetwork. Its core is to utilize the hyper network to dynamically generate the weights of the quality assessment network based on the features of the input image, thus adapting to different image contents and distortion types, which is highly adaptive and robust, capable of dealing with a wide range of distortions without the need for a reference image, and utilizes deep learning to extract features to improve the accuracy of the assessment. However, the model has high complexity and high computational resource requirements for training and inference. The model proposed by TRES-IQA [4] uses a combination of CNN and self-attention mechanism to utilize both local and non-local information of the image and fuses non-local features with local features to predict the final image quality score. It is characterized by robustness, high accuracy and flexibility. However, it has high computational complexity and complex feature transformations and fusions make the results difficult to interpret. MANIQA [17] utilizes the VIT to extract the image information followed by using the Transposed Attention Block (TAB) and the Scale Swin Transformer Block (SSTB) to use the attention block in the channel dimension, increase the interaction between local and non-local features, and finally use the dual-branching (Image Score Prediction Branch and Weighting Branch) structure to derive the final image quality score by computing the score in each patch and the product of the weights in each patch to derive the score of each patch, and finally, the scores of all the patches of the whole image are summed up to get the score of a whole image. It utilizes multi-scale features to capture image information at different scales and attention mechanism to increase robustness. However, its multi-scale feature extraction and attention mechanism increase the computation and time consumption. The specific methods are demonstrated in Table 1.

**Table 1:** Various approaches and advantages of deep learning based IQA.

	Method	Advantage
DIIVINE	Multi-scale Gabor filters and statistical features to extract image features, use SVMs to recognize distortion types, and perform quality assessment based on specific distortion types	Accurate and robust evaluation without reference images
BRISQUE	NSS model extracts spatial domain features of an image and uses SVR to predict image quality	No reference image required, high computational efficiency, stable performance
ILNIQE	Reference-free image quality assessment using the NSS model, quality prediction by extracting local features and using a Gaussian distribution model	No need for a reference image, excellent performance in a variety of distortions, high adaptability
BIECON	CNNs were applied to perform NR-IQA	Automatic extraction of image features through deep learning with high evaluation accuracy and robustness, capable of handling multiple types of image distortion
MEON	Evaluating Image Quality by Multiscale Edge Feature Networks	Capture details and edge information in images to improve the accuracy and reliability of quality assessment, especially when working with detailed images
WaDIQaM	Combining Wavelet Transform and Deep Learning to Evaluate Image Quality	Effectively captures multi-scale and multi-directional features of images, improving the accuracy and robustness of image quality assessment
DBCNN	NR-IQA with CNN	Effectively capturing image quality information through deep feature learning improves the accuracy and robustness of evaluation
TIQA	Combines time-domain features to assess image quality. Utilizes time-series information to capture dynamic changes in a video or image sequence to improve the accuracy of quality assessment	Ability to handle moving images
MetaIQA	Through meta-learning, MetaIQA can quickly adapt to new image quality assessment tasks across multiple tasks and achieve good generalization capabilities	Efficient quality assessment can be performed with a small amount of training data with good adaptability and generalizability
P2P-BM	A Patch-to-Patch matching mechanism is used to evaluate the image quality by comparing localized blocks in the image.	Capable of accurately capturing the details of localized distortions in an image with good robustness to various distortion types
HyperIQA	By using Hyper Network to generate weights for the image quality assessment model, combined with CNN to extract image features	Adaptive tuning of model parameters to improve evaluation accuracy and robustness
TReS	Extracting features for image quality assessment using a Transformer-based structure combined with a self-attention mechanism	Capable of capturing both global and local features in an image, improving the accuracy and robustness of the evaluation
MANIQA	Reference-free image quality assessment by multi-scale self-attention mechanism, extracting multi-scale features of the image to comprehensively assess the image quality	Capable of effectively capturing image information at different scales, improving the accuracy and robustness of evaluation

Currently, CNN is the main method for feature extraction in NR-IQA, although CNN has achieved good results in capturing local features of an image, it lacks the ability to capture global features of an image and has a strong local bias. Since IQA is highly dependent on global features, we use ST to extract the features of the image. The use of Vision Transformer (VIT) is proposed in MANIQA to extract the global features of the image. Unlike it we use ST to extract features.ST has lower computational complexity and higher computational efficiency compared to standard Transformer [41]. The Sliding Window Multi-Head Self Attention (SW-MSA) mechanism is used in ST. Attention is computed within a localized window, which reduces the computational complexity. VIT uses a global self-attention mechanism with higher computational complexity. Since ST computes self-attention within a local window, its computational complexity is linearly related to the image size. In contrast, VIT’s global self-attention computational complexity is square to the image size, so ST is more efficient in processing high-resolution images.

### Attention mechanism

Attention mechanisms are widely used in various computer vision tasks [50–54]. Attention Mechanism in Deep Learning is an approach that mimics the human visual and cognitive system by allowing neural networks to focus on relevant parts of the input data as it is processed. By introducing the Attention Mechanism, neural networks can automatically learn and selectively focus on important information in the input, improving the performance and generalization of the model. Self-attention is a commonly used attention mechanism in deep learning, The basic idea of the self-attention mechanism is that when dealing with sequential data, each element can be associated with other elements in the sequence, rather than just relying on elements in neighboring positions. It adaptively captures long-range dependencies between elements by calculating their relative importance.

However, conventional Self-attention ignores the rich information between different channels. This may lead to an inadequate feature representation that fails to adequately capture the complex feature relationships in the image, and can result in the loss of contextually important information. In NR-IQA, Restormer [54] applies the self-attentional mechanism in a novel way by exchanging the positions of spatial and channel dimensions to reduce computation and implicitly fuse global features. MANIQA proposed an innovation in the attention mechanism using channel direction when using SA, which greatly improved the accuracy of the model. Inspired by the above, we propose GSAB to capture feature dependencies, fusing information of spatial and channel concerns with each other in order to capture richer and more comprehensive features and improve the expressiveness of the model and the accuracy of the prediction.

### Proposed method

**Fig 1:**
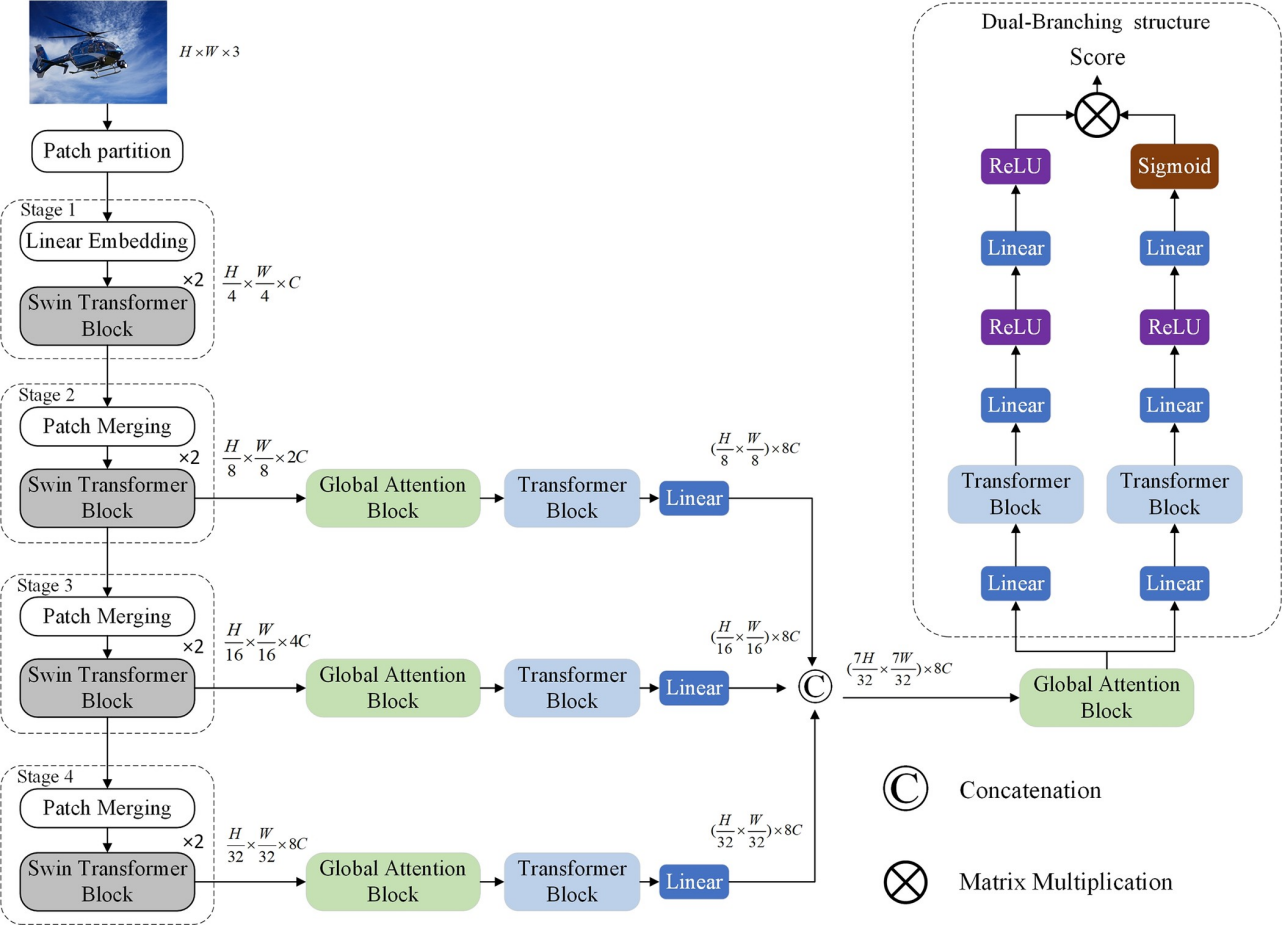
No-reference image quality assessment based on ST. The distorted image is fed into the Swin Transformer (Fig 2) for feature extraction. ‘Global Self-Attention Block’ details the information interaction between the global self-attention block space and the channel dimensions. ‘Quality prediction block with dual-branching structure’ details how the two-branch structure can be used for quality prediction.

In this study, we design a model that can extract multidimensional image features, which consists of four parts: as shown in Fig 1 (A): Swin Transformer, (b): Transformer Block, (c): Global Self-Attention Block, (d): Transformer Block-based dual-branch structure for quality prediction.

**Fig 2:**
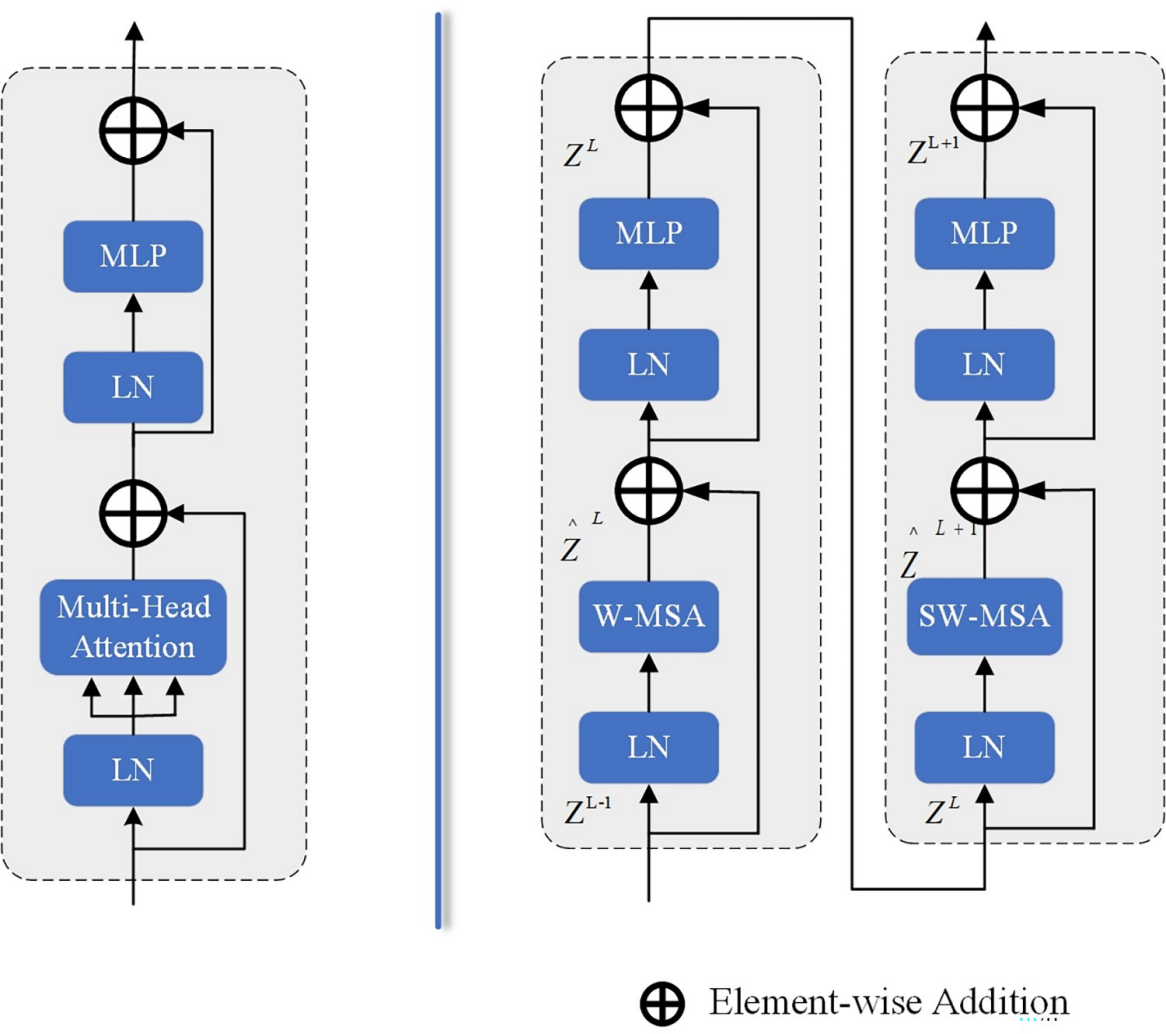
Left: Transformer Block, right: Swin Transformer Block. W-MSA is multi-head self-attention modules with regular configuration and SW-MSA is multi-head self-attention modules with shifted windowing configuration. LN is Layernorm.

### Overall modeling framework

Input an image *I*∈*R*^*H*×*W*×3^, here H and W represent the height and width and our goal is to evaluate the prediction score (q) of the image. $F_{i}\in R^{b\times h_{i}\times w_{i}\times c_{i}}$ represents the features from the *i*_*th*_ layer of Swin Transformer, here *i*∈{1,2,3,4}, b represents the Batch Size, and *h*_*i*_, *w*_*i*_ and *c*_*i*_ represents the feature’s height, width, and channel size, respectively. To enhance the representation of the model we use multi-scale features for fusion, but to reduce the computational complexity of the model, we only use the features extracted from stage 2, stage 3, and stage 4 in Swin Transformer to feed into the GSAB and the TB to enhance the channel and spatial feature extraction capability. Then the obtained features are fused. The final fused feature maps will be fed into the TB based dual-branching structure, which has two components, image quality score prediction, and weighted prediction, and the final image prediction scores can be obtained by the sum of the products of the scores and weights of each patch. Eq (1) shows how fractions are calculated:

$$q=\frac{\sum_{i=0}^{N}w_{i}\times s_{i}}{\sum_{i=0}^{N}w_{i}}$$ (1)

Where N represents the number of patches in an image, *i* represent the layer of the ST, w represents the weight, and s represents the score.

### Global self-attention block

Self-Attention [16] is the core of the Transformer, which can be regarded as a feature extraction layer, where a given input feature is fused with each input feature by the Self-Attention Layer to get a new feature. Its input can be the original Input or the hidden layer. In self-attention, each Input is multiplied by 3 different Matrice to produce 3 different Vectors named q, k, and v, respectively. Self-Attention multiplies the input features by three matrices *W*^*q*^, *W*^*k*^, and *W*^*v*^ (weight matrices of q, k, v respectively) to get three matrices Q (Query), K (Key), and V (Value). Next, the product of the matrices Q and K is used to divide by the dimension of the matrices, and finally, the Soft-max computed values are multiplied by V to get the final output features. Eq 2 shows the calculation of Self-Attention:

$$Attention(Q,K,V)=soft\max(\frac{QK^{T}}{\sqrt{d_{k}}})V$$ (2)

Where Q represents the Query, K represents the Key, and V represents Value.

**Fig 3:**
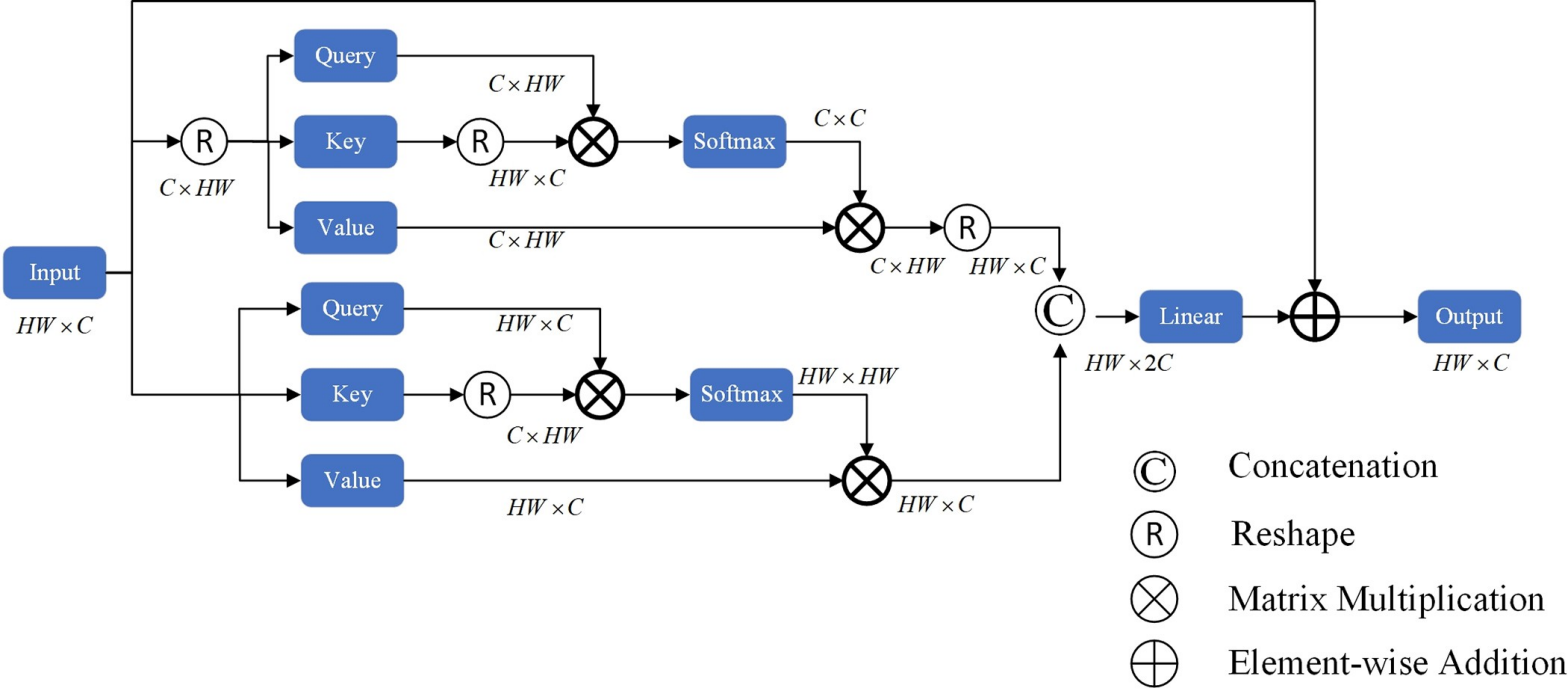
Global self-attention block.

In traditional Self-Attention, the main focus is on spatial connections, but the connections on channels are neglected. To enable the effective application of both spatial and channel-based features, we propose the Global Self-Attention Block as shown in Fig 3. Specifically, we incorporate Self-Attention applied across channels while preserving global connections between patches in the spatial dimension has generated implicitly encoded attention graphs for global contextual questions. Our global self-attention module, without increasing the spatial and channel dimension bars, pays attention to the spatial information as well as the information of the channel, which achieves the effect of taking into account the global situation.

### Quality prediction block with dual-branching structure

In MANIQA [17], it is introduced that traditional score prediction is prone to overfitting, to solve this problem we use a dual-branching structure for score prediction, and we are inspired to improve the dual-branching structure based on this. The improved module consists of two parts, the score prediction branch and the weighting branch, and in order to improve the prediction performance of the model and to enable the establishment of dependencies between different feature layers after fusion, we add one TB in each branch of the module. The patch prediction score of the final image is obtained by multiplying the score and weight of each patch, and the final prediction score of the whole image is obtained by summing the scores of each patch.

## Experiments

### Datasets

The LIVE [9] dataset is the largest available annotated image quality dataset, the LIVE dataset consists of 799 images, the dataset uses five computer distortion operations to degrade the quality of the reference image by 5–6 levels respectively, resulting in 779 distorted images, and the image size is mostly 768×512. There are five main types of these distortions, namely: JPEG2000 compression, JPEG compression, White Noise, Gaussian Blur, Simulated Fast Fading Rayleigh (wireless) Channel. JPEG2000 compression is to use JPEG2000 to compress the reference image at a bit rate of 0.028 bits per pixel (bpp) to 3.15bpp to generate a distorted image; JPEG compression is to use JPEG to compress the reference image at a bit rate of 0.15bpp to 3.34bpp. White Noise is to add standard Gaussian white noise to the RGB components of the image, and the resulting distorted components are clipped between 0 and 1 and rescaled to the range of 0 to 255.Gaussian Blur is to add a standard Gaussian white noise to the RGB components of the image after the R, G, and B components were filtered using a circular-symmetric 2-D Gaussian. Simulated Fast Fading Rayleigh (wireless) Channel: Images were distorted by bit errors during transmission of compressed JPEG2000 bitstream over a simulated wireless channel. Receiver SNR was varied to generate bitstreams corrupted with different proportion of bit errors.

The CSIQ [11] dataset was created in 2009 and contains 30 original images and 866 synthetic distorted images. Each distortion type was subjected to a quality reduction operation at 4 to 5 different distortion levels to obtain 866 distorted versions of the original image. 5000 DMOS evaluation data were made by 25 volunteers for the CSIQ dataset. There are six main types of distortion used, which are: JPEG compression, JPEG-2000 compression, global contrast decrements, additive pink Gaussian noise, additive white Gaussian noise, and Gaussian blur. global contrast decrements: generate distorted images by reducing the overall contrast of the image and narrowing the brightness range of the image. additive pink Gaussian noise: is generated by converting the original image with Gaussian white noise added to it to pink noise by applying a pink noise filter. additive white Gaussian noise: is generated by generating a normally distributed random noise process with zero mean and unit variance, scaling the noise according to the desired standard deviation, and finally adding the scaled noise to the original signal.

The basic requirement for datasets designed and evaluated by HVS metrics is that the IQA dataset should contain a large number of standard color images of various contents. To satisfy this requirement the TID2013 [10] dataset created in 2013 contains 3000 images obtained from 25 reference images, each of which contains 24 distortion types, with each distortion type classified into 5 levels.

Current databases for evaluating the quality of artificially distorted images are small and limited in content. In order to promote IQA, Konstanz University created the KADID [7] dataset. KADID is a collection of raw images with a resolution greater than 1500×1200 from Pixabay.com, were rescaled to the same resolution as TID2013 (512×384) while maintaining the pixel aspect ratio, cropping was performed if needed. Finally, 81 high-quality images were manually selected as raw images for KADID-10k, and each raw image was degraded with 25 distortions at 5 levels.

Most of the existing datasets are synthesized in the laboratory by adding various noises to the original images, but for real world multiple graphical distortions are not necessarily well modeled by the existing databases. So, Deepti Ghadiyaram and Alan C. Bovik built the LIVE In the Wild Image Quality Challenge Database (CLIVE) [13] dataset in 2016. This dataset contains a variety of real image distortions from a large number of images captured using a variety of representative modern mobile devices. Subjective scores were collected through crowdsourcing and evaluated by more than 8,100 testers, and more than 350,000 subjective opinion scores were collected for the 1,162 images, with MOS values ranging from [3.42–92.43].

Deep learning in IQA is limited by the small size of existing datasets. With this in mind, Vlad Hosu established the KonIQ-10k [14] dataset, the largest field database to date, in 2020.KonIQ-10k selects 10,073 images from the YFCC100M dataset (a large, public multimedia dataset containing 10 million images), and the sampling process utilizes a depth-feature-based content metric and seven quality metrics to ensure a diverse distribution of image content and quality, allowing for a broad and even distribution of images in terms of brightness, color, contrast, and sharpness.

In the above six datasets, LIVE, CSIQ, TID2013, and KADID are distorted images synthesized in the laboratory by adding various kinds of noises to the images, while CLIVE and KonIQ-10k are real distorted images obtained by shooting in natural scenes.

Table 2 shows the details of each dataset.

**Table 2:** Summary of IQA datasets.

Dataset	# of Dist.	# of Dist.	Distortions
	Images	Types	Type
LIVE [9]	799	5	synthetic
CSIQ [11]	866	6	synthetic
TID2013 [10]	3000	24	synthetic
KADID [12]	10125	25	synthetic
CLIVE [13]	1162	-	authentic
KonIQ-10k [14]	10073	-	authentic

### Assessment criteria

To evaluate the accuracy of image quality prediction, we introduced Spearman’s rank order correlation coefficient (SROCC) and Pearson’s linear correlation coefficient (PLCC) to assess our model predicted quality scores. SROCC has a part of the literature written as SRCC, which is used to measure the monotonicity of the prediction of the IQA algorithm. The calculation is shown in Eq 3:

$$SRCC=1-\frac{6\sum_{i=1}^{N}d_{i}^{2}}{N(N^{2}-1)}$$ (3)

Where N represents the number of samples and *d*_*i*_ represents the difference between the subjective quality score ranking of the *i*_*th*_ image and the objective quality score ranking.

SROCC on the rank size of the two target arrays for linear correlation analysis (rank correlation of the two sets of data), often considered to be the PLCC of the two objects respectively after the arrangement, the scope of application is relatively broad. The range of values is [–1,1], the larger the absolute value, the better.

The Pearson Linear Correlation Coefficient (PLCC) is used to assess the accuracy of the predictions of the IQA model. The PLCC evaluates the correlation between the MOS correlation with the objective scores after nonlinear regression. Before calculating the PLCC, a nonlinear regression operation (nonlinear fitting) is required to establish a nonlinear mapping between the objective scores and the subjective scores. Eq 4 calculates the PLCC:

$$PLCC=\frac{\sum_{i=1}^{N}\left(s_{i}-\bar{s}\right)(p_{i}-\bar{p})}{\sqrt{\sum_{i=1}^{N}{\left(s_{i}-\bar{s}\right)}^{2}\sum_{i=1}^{N}{\left(p_{i}-\bar{p}\right)}^{2}}}$$ (4)

Where *s*_*i*_ and *p*_*i*_ denote the subjective quality score and objective quality score of the *i*_*th*_ image.

PLCC describes the linear correlation between the two data, and its value range is [–1,1]. When the value of PLCC is zero, it indicates that the two data are not correlated at all (the objective and subjective quality scores of the images are very different); when the value of PLCC is 1 or -1, it indicates that the two data are correlated at all (the objective quality score of the image is the same as the subjective quality score). The PLCC describes the correlation between the algorithm’s objective assessment scores and the subjective scoring by the human eye and measures the IQA algorithm’s predictive Accuracy.

### Implementation details

Our experiments were conducted on an NVIDIA RTX4090 with PyTorch using version 2.1.2 and CUDA using version 12.1 for training and testing. We conducted our experiments on six datasets containing two categories, authentic and synthetic datasets. Following the standard strategy for IQA training, we took each image selected 25 blocks of 224×224 patches, and randomly flipped them horizontally and vertically for enhancement. We used L1 loss during training:

$$L_{1}=\sum_{i=1}^{n}\left|y_{i}-f(x_{i})\right|$$ (5)

Where *y*_*i*_ and *f*(*x*_*i*_) denote the true score value of the image and the score value predicted by the model.

We used Adam [55] optimizer with weight decay 5×10^−4^ to train our model for at most 10 epochs, with a batch size of 24, and the learning rate was set to 2×10^−5^, and in the testing phase, we sampled each image with a size of 224 × 224 of 10 image blocks for each image and the final score is the average of the scores of these ten image blocks.

Following the IQA standard training method, we used six datasets for training and randomly divided the images of each dataset into 8:2 for training and testing, ten times randomly, so we have ten different training and testing sets. The dataset of the test set was not used in the training. For all the results in this paper, we conducted ten experiments with different training and test sets and finally took the median of SRCC and PLCC respectively as the final experimental results.

## Results

### Accuracy experiments

**Fig 4:**
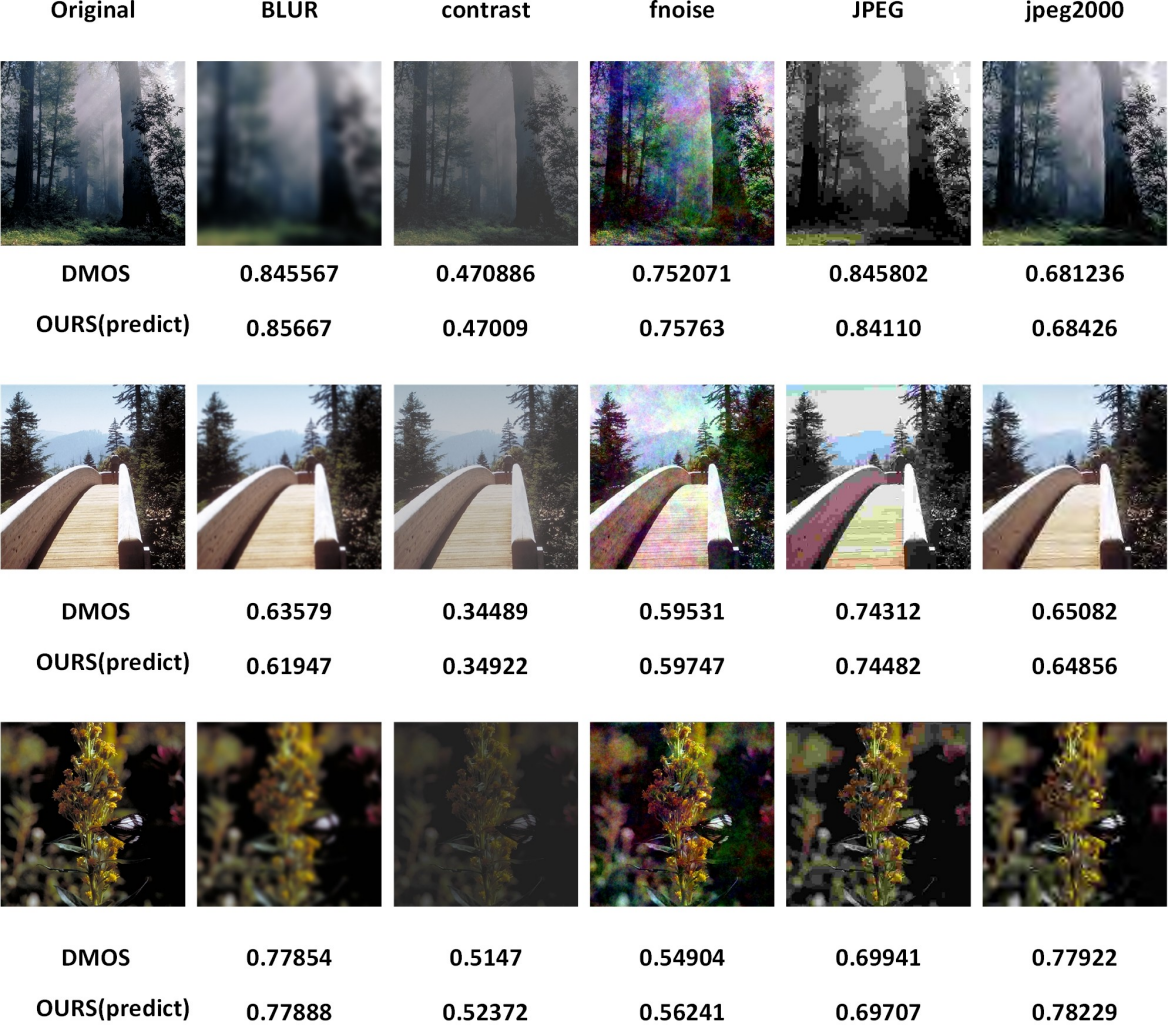
Example image from CSIQ [11] dataset. On the left is the original image on the right is the distorted image, below are the image manual evaluation scores DMOS and our model predicted scores respectively, it can be observed that our model has high accuracy.

To verify the accuracy of our model, we selected three groups of images with five distortion types in the CSIQ dataset, the distortion types are Gaussian blur (BLUR), global contrast decrements(contrast), foveated noise(fnoise), JPEG compression (JPEG), JPEG-2000 compression(jpeg2000). The first column is the undistorted original image, and each column represents one type of distortion, the CSIQ label is DMOS. We find the value of DMOS for each distorted image and use our model to predict the value of the related image for comparison, from Fig 4, we can see that our error rate is around 0.1%-0.8% for BLUR distortion, 1% for contrast distortion, 0.5%-2% for fnoise distortion, 0.5%-2% for fnoise distortion, JPEG distortion the error rate is 0.1%-0.4%, and in jpeg2000 distortion type the error rate is 0.3%-0.6%. From the above results, it can be seen that the error rate of our model is roughly maintained below 1%, which also proves the accuracy of our model prediction.

### Experimental linearity

To verify the linearity of our model, we perform experiments on the CSIQ and KonIQ-10k datasets. And the model was subjected to fit accuracy R^2^. R^2^ is a statistical metric used to evaluate the performance of the model. The value of R^2^ ranges from 0–1, when equal to 1 it means that the model explains all the variations in the data perfectly and 0 means that it fails to explain any of the data variations.

$$R^{2}=1-\frac{\sum {\left(y_{i}-y_{j}\right)}^{2}}{\sum {\left(y_{i}-\bar{y}\right)}^{2}}$$ (6)

*y*_*i*_ is the tag score value of the picture, *y*_*j*_ is the fraction predicted by our model, $\bar{y}$ is the mean of the label values.

**Fig 5:**
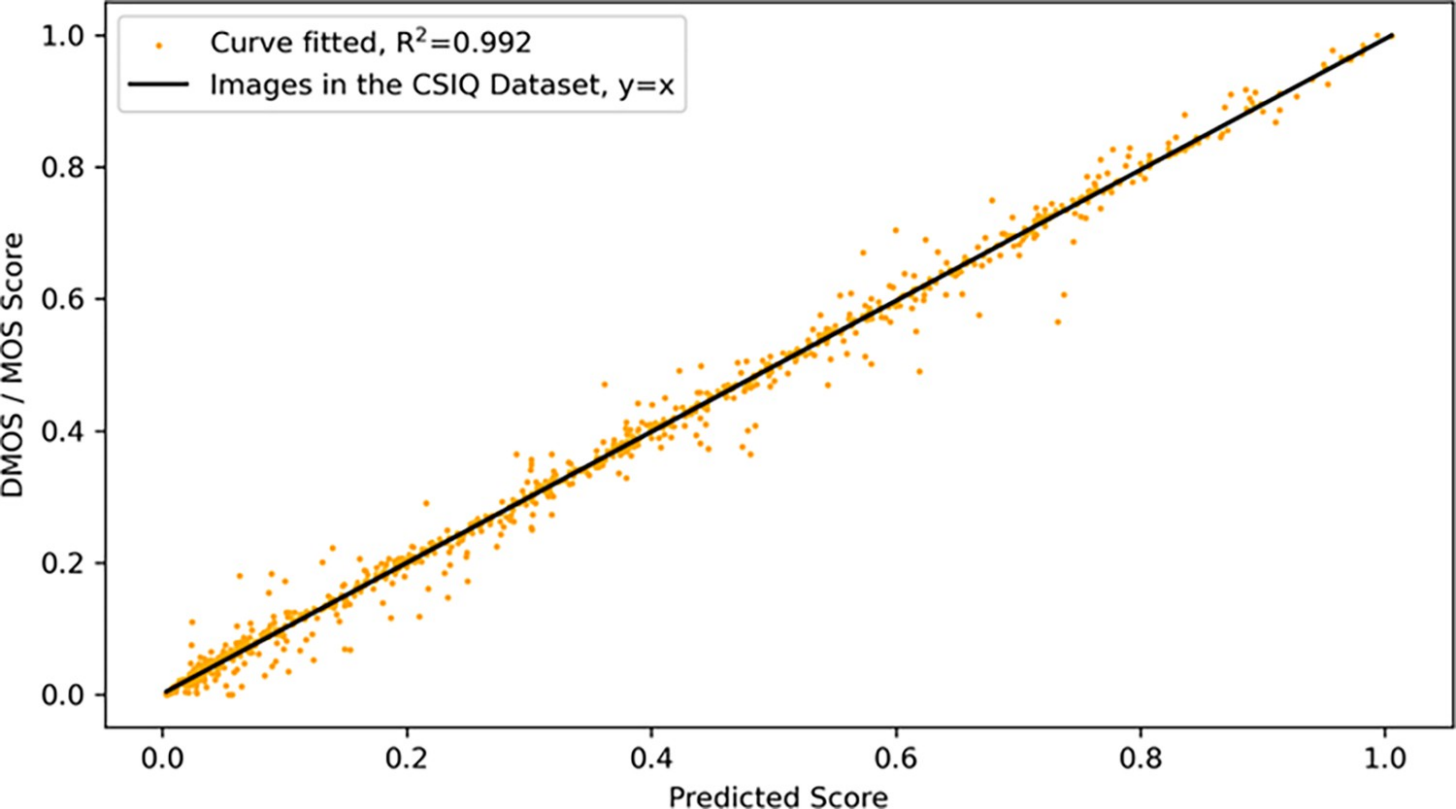
Scatterplot of the CSIQ dataset and regression curves with predicted scores in the horizontal coordinate and DMOS values subjectively observed by the human eye in the vertical coordinate.

Fig 5 shows the linearity curves on the CSIQ dataset, which is a small dataset of distorted images synthesized by a laboratory based on the original images with various types of distortion processing, and then averaged according to the subjective judgmental score values observed by the human eye to obtain the value of DMOS. The black line in Fig 5 is y = x. The yellow dot is the intersection between the image score predicted by our model and the DMOS value, and when the predicted value is exactly equal to the DMOS value, the dot falls on the line y = x. It can be observed that the R^2^ of our model can reach 0.992, which also proves that our model has a good linearity.

**Fig 6:**
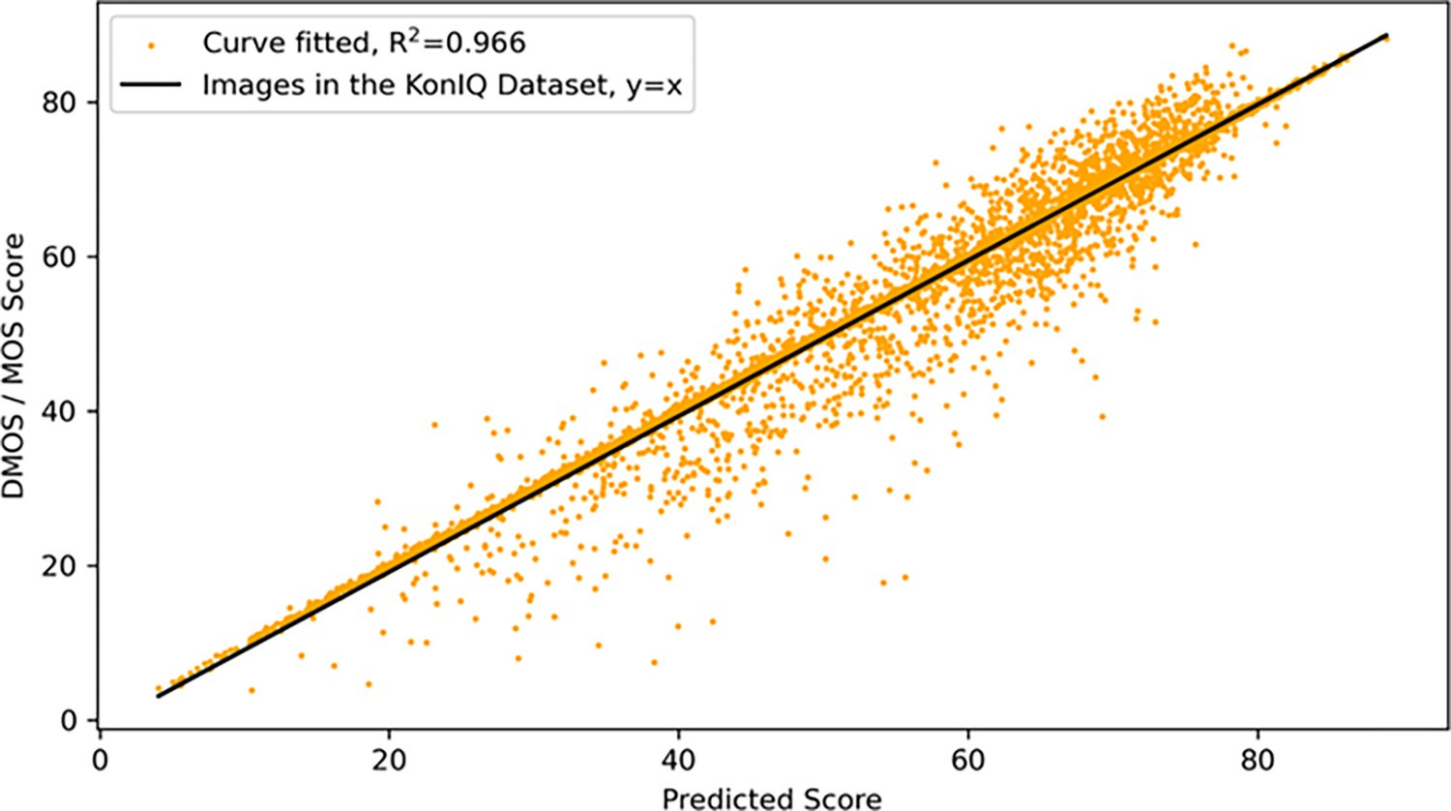
Scatterplot of the KonIQ-10k dataset and regression curves with predicted scores in the horizontal coordinate and MOS values subjectively observed by the human eye in the vertical coordinate.

In order to verify the linearity of our model on different types and sizes of data sets, we performed validation on the KonIQ-10k dataset, which is a large dataset of naturally distorted images. Fig 6 shows the results of our experiment on the KonIQ-10k dataset. The vertical coordinate is the MOS value evaluated by subjective observation of human eyes, and the horizontal coordinate is the fractional value predicted by our model. As can be seen from Fig 6, our scatter plot is closely concentrated near our regression curve, and our R^2^ value is 0.966, which also proves the good linearity of our model.

From the above results, it can be seen that our model has good linearity on both synthetic and authentic datasets. This also proves that our model has stable results for different types and sizes of datasets, and thus proves the reliability of the model.

### Assessment on six datasets

Tables 3 and 4 show the comparison of the overall performance of SRCC and PLCC on six different standard image datasets, which contain both authentic image datasets and synthetic image datasets. Our model outperforms the existing methods on most of the datasets and especially shows better results on the authentic image dataset. The performance of our model not only improves significantly on large datasets but also achieves competitive results on small datasets. In the last column, we provide the average weighted performance for all datasets, weighted according to the number of images in each dataset as weights. It can be observed that our method outperforms all existing methods.

**Table 3:** SRCC performance assessment on six image databases. Black and blue fonts indicate best and second-best performance, respectively. Some data from [4].

SRCC	LIVE	CSIQ	TID2013	KADID	CLIVE	KONIQ	Average
DIIVINE [6]	0.892	0.804	0.643	0.413	0.588	0.546	0.527
BRISQUE [5]	0.929	0.812	0.626	0.528	0.629	0.681	0.625
ILNIQE [8]	0.902	0.822	0.521	0.534	0.508	0.523	0.548
BIECON [56]	0.958	0.815	0.717	0.623	0.613	0.651	0.661
MEON [38]	0.951	0.852	0.808	0.604	0.697	0.611	0.653
WaDIQaM [35]	0.960	0.852	0.835	0.739	0.682	0.804	0.783
DBCNN [42]	0.968	0.946	0.816	0.851	0.869	0.875	0.864
TIQA [57]	0.949	0.825	0.846	0.850	0.845	0.892	0.868
MetaIQA [43]	0.960	0.899	0.856	0.762	0.835	0.887	0.835
P2P-BM [58]	0.959	0.899	0.862	0.840	0.844	0.872	0.861
HyperIQA [39]	0.962	0.923	0.840	0.852	0.859	0.906	0.878
TReS [4]	**0.969**	0.922	0.863	0.859	0.846	0.915	0.886
Ours	0.964	**0.963**	**0.881**	**0.933**	**0.874**	**0.923**	**0.922**

**Table 4:** PLCC performance assessment on six image databases. Black and blue fonts indicate best and second-best performance, respectively. Some data from [4].

PLCC	LIVE	CSIQ	TID2013	KADID	CLIVE	KONIQ	Average
DIIVINE [6]	0.908	0.776	0.567	0.435	0.591	0.558	0.531
BRISQUE [5]	0.944	0.748	0.571	0.567	0.629	0.685	0.633
ILNIQE [8]	0.906	0.865	0.648	0.558	0.508	0.537	0.579
BIECON [56]	0.961	0.823	0.762	0.648	0.613	0.654	0.677
MEON [38]	0.955	0.864	0.824	0.691	0.710	0.628	0.697
WaDIQaM [35]	0.955	0.844	0.855	0.752	0.671	0.807	0.791
DBCNN [42]	**0.971**	0.959	0.865	0.856	0.869	0.884	0.875
TIQA [57]	0.965	0.838	0.858	0.855	0.861	0.903	0.877
MetaIQA [43]	0.959	0.908	0.868	0.775	0.802	0.856	0.828
P2P-BM [58]	0.958	0.902	0.856	0.849	0.842	0.885	0.869
HyperIQA [39]	0.966	0.942	0.858	0.845	0.882	0.917	0.883
TReS [4]	0.968	0.942	0.883	0.858	0.877	0.928	0.895
Ours	0.966	**0.969**	**0.898**	**0.935**	**0.893**	**0.935**	**0.931**

### Generalization ability test

In Table 5, we performed a Generalization ability test to compare our model with competing approaches. The training was performed on one dataset and testing was performed on the other dataset without any tuning of the parameters. Compared to other methods, our proposed method has better results, which also proves that our model has better generalization.

**Table 5:** SROCC assessments on cross datasets, black and blue are the best and second-best performers.

Train	LIVEC	KONIQ	LIVE	
Test	KONIQ	LIVEC	CSIQ	TID2013
WaDIQaM [35]	0.711	0.682	0.704	0.462
DBCNN [42]	0.754	0.755	0.758	0.524
P2P-BM [58]	0.740	0.770	0.712	0.488
HyperIQA [39]	**0.772**	0.785	0.744	0.551
TReS [4]	0.733	0.786	0.761	0.562
OURS	0.768	**0.797**	**0.765**	**0.585**

### Ablation study

To evaluate the effect of our proposed components, we conducted an Ablation Study on CSIQ and KADID datasets, we first utilized ST as our backbone model and analyzed the impact of each component by SRCC and PLCC.

**Fig 7:**
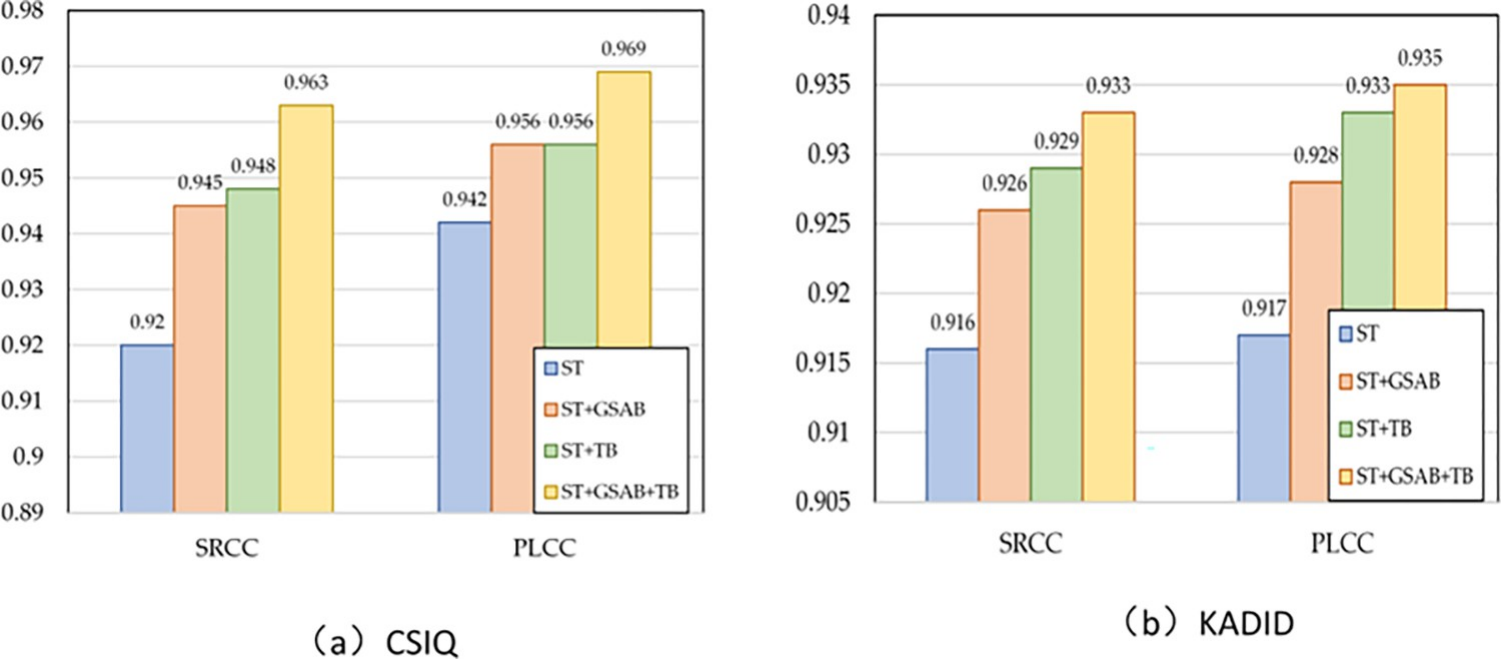
Ablation results on CSIQ and KADID.

First, we combine ST and GSAB to test the effectiveness of our proposed Global Self-Attention mechanism. Experimentally, both SRCC and PLCC of the test results show significant improvement on both CSIQ and KADID datasets. Then, the TB is added to the backbone network, and after testing, the performance on both our CSIQ and KADID databases is significantly improved after adding TB compared to the backbone-only case. Finally, we combine all the components to get the final result. After the experiments, SRCC and PLCC are significantly improved on both databases, From Fig 7(A), it can be seen that the value of SRCC for CSIQ has increased by 4.3% and the value of PLCC for CSIQ has increased by 2.7%. From Fig 7(B), it can be seen that the value of SRCC for KADID has been improved by 1.7% and the value of PLCC for KADID has been improved by 1.8%.

To verify the effectiveness of our improved global attention module, we subjected MANIQA’s attention module to the same treatment as ours under the same experimental conditions. The SRCC and PLCC of the final six data sets are obtained. The data from Table 6 shows that our attention module has better performance.

**Table 6:** MANIQA’s attention module compared with our GSAB results on six datasets.

	MANIQA		GASB	
	SRCC	PLCC	SRCC	PLCC
LIVE	0.955	0.956	0.964	0.966
CSIQ	0.958	0.966	0.963	0.969
TID20133	0.895	0.908	0.881	0.898
KADID	0.923	0.924	0.933	0.935
CLIVE	0.862	0.881	0.874	0.893
KonIQ-10k	0.918	0.929	0.923	0.935

### Visualization

**Fig 8:**
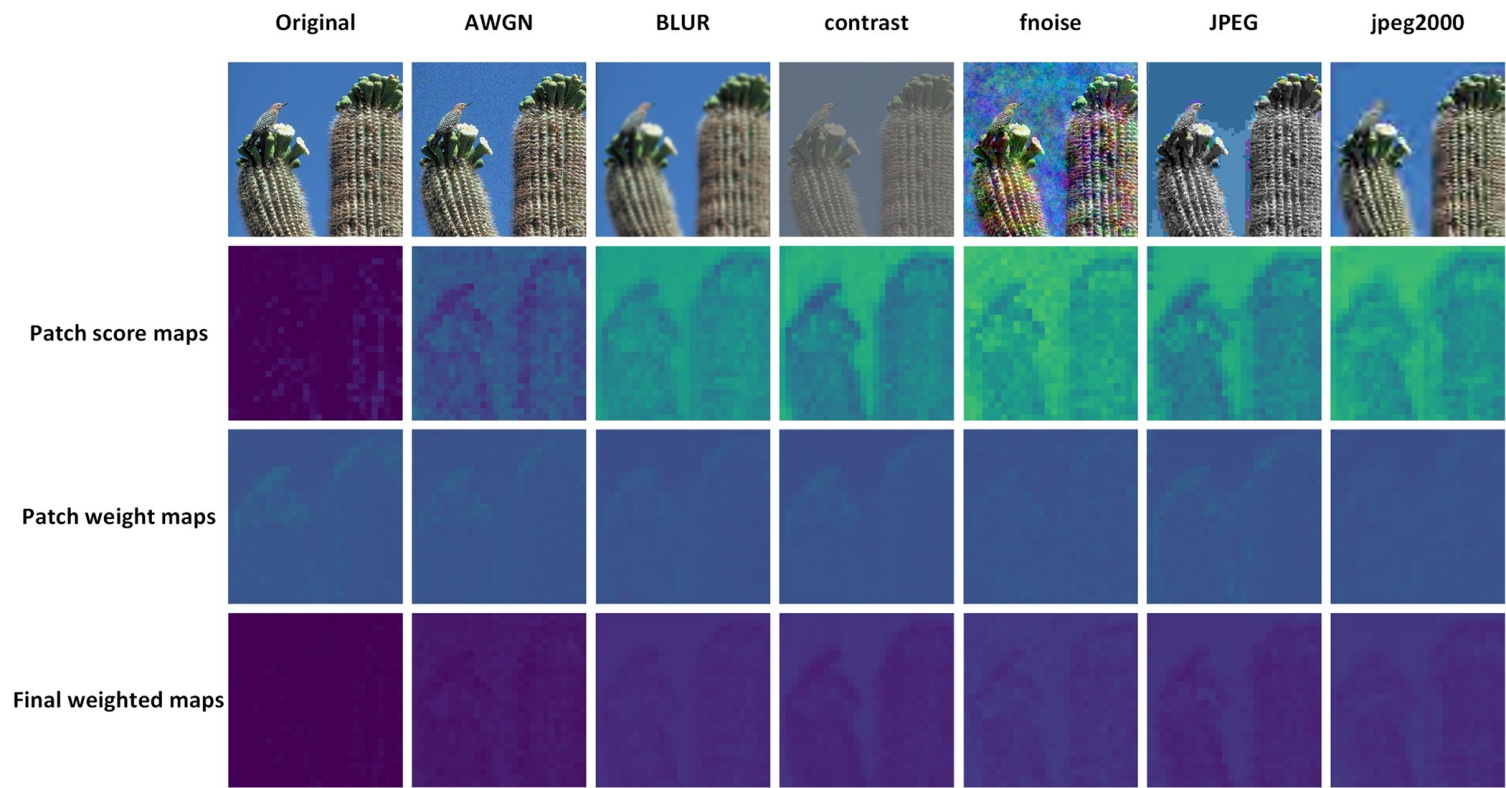
Visualization of patch score maps, patch weight maps and final weighted maps of the dataset of the CSIQ [11].

In Fig 8, we visualize the patch scores, patch weights, and final weighted maps on the CSIQ dataset. We selected six different distortion types of the same image for visualization. The score maps highlight regions with better visual experience, while the weight maps focus on areas that are significant to the human eye. From the images of different distortion types, the score maps and weight maps vary, demonstrating that our model can distinguish between different types of distortions, thereby proving its reliability. For the same type of distortion, different noise levels are reflected in the score maps with different colors. In the case of the original image, which we consider to have almost no noise, the final score map shows relatively uniform colors, indicating nearly equal weight distribution. However, in distorted images, we assign different weights based on the key focus areas in the score map. This allows our model to predict different quality scores for images with varying levels of distortion.

## Conclusions

In this paper, we use a new deep learning model instead of manual feature extraction to address the quality assessment of authentic images. Our model uses the Swin Transformer as the main framework and adds a Global Self-Attention Block and Transformer Block to enable more spatial and on-channel information to be attended to, and in image quality score prediction, we perform image score prediction in the addition of a dual-branching structure capable of establishing remote dependencies in the Prediction. The experiments show that our proposed method far outperforms all existing methods on authentic image datasets and also gives better results on synthetic datasets. In the Generalization ability test, our method outperforms existing methods on most datasets, which also proves the good generalization ability of our model.
